# Notch signaling indirectly promotes chondrocyte hypertrophy via regulation of BMP signaling and cell cycle arrest

**DOI:** 10.1038/srep25594

**Published:** 2016-05-05

**Authors:** Xifu Shang, Jinwu Wang, Zhengliang Luo, Yongjun Wang, Massimo M. Morandi, John V. Marymont, Matthew J. Hilton, Yufeng Dong

**Affiliations:** 1 Department of Orthopedic Surgery, Anhui Provincial Hospital, 17 Lujiang Rd, Hefei, China; 2 Shanghai Key Laboratory of Orthopedic Implant, Department of Orthopaedic Surgery, Shanghai Ninth People’s Hospital Affiliated Shanghai Jiao Tong University School of Medicine, Shanghai, China; 3 Institute of Spine, Longhua Hospital, Shanghai University of Traditional Chinese Medicine, Shanghai, China; 4 Department of Orthopedic Surgery, Louisiana State University Health Sciences Center, Shreveport, Louisiana, USA; 5 Departments of Orthopedic Surgery and Cell Biology, Duke Orthopedic Cellular, Developmental, and Genome Laboratories, Duke University School of Medicine, Durham, North Carolina, USA

## Abstract

Cell cycle regulation is critical for chondrocyte differentiation and hypertrophy. Recently we identified the Notch signaling pathway as an important regulator of chondrocyte proliferation and differentiation during mouse cartilage development. To investigate the underlying mechanisms, we assessed the role for Notch signaling regulation of the cell cycle during chondrocyte differentiation. Real-time RT-PCR data showed that over-expression of the Notch Intracellular Domain (NICD) significantly induced the expression of *p57*, a cell cycle inhibitor, in chondrocytes. Flow cytometric analyses further confirmed that over-expression of NICD in chondrocytes enhances the G0/G1 cell cycle transition and cell cycle arrest. In contrast, treatment of chondrocytes with the Notch inhibitor, DAPT, decreased both endogenous and BMP2-induced SMAD 1/5/8 phosphorylation and knockdown of SMAD 1/5/8 impaired NICD-induced chondrocyte differentiation and *p57* expression. Co-immunoprecipitation using p-SMAD 1/5/8 and NICD antibodies further showed a strong interaction of these proteins during chondrocyte maturation. Finally, RT-PCR and Western blot results revealed a significant reduction in the expression of the SMAD-related phosphatase, PPM1A, following NICD over-expression. Taken together, our results demonstrate that Notch signaling induces cell cycle arrest and thereby initiates chondrocyte hypertrophy via BMP/SMAD-mediated up-regulation of *p57*.

During skeletal development, cartilage is derived from condensed mesenchyme tissue, which involves proliferation and differentiation of mesenchymal progenitor cells into round immature chondrocytes, flattened mature chondrocytes or pre-hypertrophic chondrocytes and ultimately hypertrophic chondrocytes^1^,^2^.

A number of studies have shown that cell cycle arrest is required for chondrocyte transition from proliferation to hypertrophic differentiation because terminal differentiation is usually coupled with cell cycle exit and maintenance of the non-proliferative state^3^,^4^,^5^. The decision by a cell to continue proliferating or to exit the cell cycle for differentiation is often made during the G0/G1 phase of the cell cycle. In this phase, signals affecting cellular proliferation (e.g. growth factors, nutrients) are integrated to determine whether the cell continues to cycle or withdraws from the cell cycle via regulation of multiple proteins, including cyclins, cyclin-dependent kinases (CDK), and CDK inhibitors (CDKI), which block kinase activity and cell-cycle progression^6^,^7^. Numerous signaling pathways have been identified that regulate both cell cycle and chondrocyte hypertrophy, including BMP, TGF-β and Wnt. Recently, we identified the Notch signaling pathway as a critical regulator of chondrocyte hypertrophy, although it appears to regulate this process in an indirect manner^8^,^9^,^10^,^11^.

Notch signaling is initiated through the interaction of Notch cell-surface receptors with cell-surface ligands of the Jagged/Delta family. The mammalian family of Notch receptors consists of four members: Notch1–4, while the family of ligands consists of five members: Jagged 1, 2, Delta like 1 (Dll1), Delta-like 3 (Dll3) and Delta-like 4 (Dll4)^12^. In the absence of ligands, Notch receptors are inactive whereas binding of the ligand to the Notch receptor induces site-specific cleavages ultimately resulting in the release of the Notch Intracellular Domain (NICD). This NICD translocates to the nucleus where it modulates gene expression through interaction with members of the CSL (CBF-1, Suppressor of Hairless, and Lag-1) family of transcription factors. Notch activation leads to elevated expression of specific genes including the HES and HEY family of transcription factors^13^. The association of Notch signaling with the cell cycle has been well documented in cancer cells, including multiple myeloma, pancreatic cancer, and cancers of prostate, cervix, colon, lung, skin, and brain^14^,^15^,^16^. However, Notch regulation of cell cycle progression in chondrocytes has not been extensively studied.

To gain further mechanistic insights into how Notch signaling regulates the proliferation and differentiation of chondrocytes, and with the knowledge that BMP signaling is also involved in this function, here we investigate the potential cross talk between the two signaling cascades. In this study, primary mouse chondrocytes and the chondrogenic cell line ATDC5 were used to evaluate the effects of Notch signaling on cell cycle progression during chondrocyte differentiation with treatment of recombinant BMP2 protein. Our results demonstrate that Notch signaling induces G0/G1 arrest and subsequent differentiation of chondrocytes through increased expression of *p57*, which is also mediated by BMP-specific SMAD1/5/8 signaling.

## Results

### Notch activation promotes cell cycle exit and subsequent chondrocyte hypertrophy

**Figure 1:**
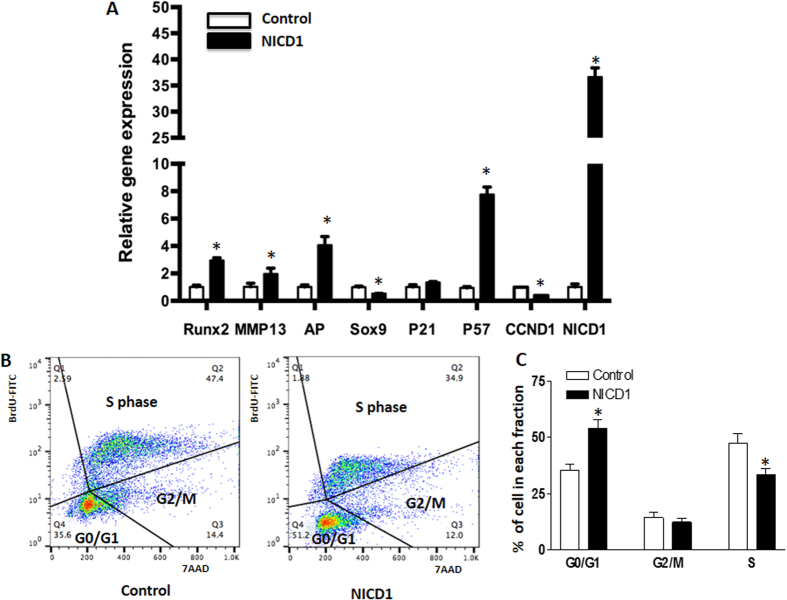
Notch activation promotes chondrocyte differentiation in ATDC5 cells by enhancing cell cycle exit. ATDC5 cells were transfected with NICD1 plasmids for 48 hours. Cells were then harvested for total RNA isolation and RT-PCR analysis. (**A**) Notch activation by NICD1 significantly increased NICD1, Runx2, MMP13, AP, and P57 gene expression and decreased Sox9 and Cyclin D1 (CcnD1) gene expression in culture. (**B**) Represented figure for cell cycle analysis. Cell population in S phase was largely reduced, and G0/G1 phase cell population was significantly increased at 48 hours post NICD1 transfection when compared to control cells. The cell cycle status was determined based on BrdU incorporation and staining with the DNA dye 7-AAD, and distribution in percentages are labeled in each dot plot. (**C**) Quantization of cell population in G0/G1, G2/M and S phase versus total cell population. PCR data are means ± SD of three independent experiments performed in duplicate and the control gene expression level was set at 1. (*p < 0.05 compared with control at same time point).

Prior work demonstrated that activation of Notch signaling by NICD promotes chondrocyte hypertrophy *in vitro* and *in vivo*^8^. Here we set out to identify potential targets involved in this regulation. First, we transfected the chondrogenic cell line ATDC5 with NICD1 expressing plasmids. Forty-eight hours post-transfection, cells were harvested for RNA isolation and real-time RT-PCR analysis was performed. These data showed a 35-fold increase in the expression of *Notch1* within cells transfected with NICD1 plasmids when compared to control cells transfected with empty vectors (Fig. 1A), thereby confirming successful transfection. We also observed a significant decrease in the expression of the early chondrogenic marker, *Sox9*, in NICD1 expressing cells, and in contrast, regulators and markers of chondrocyte hypertrophy, *Runx2, Alp* and *Mmp13*, were all up-regulated. These data suggest that Notch activation promotes a mature or hypertrophic chondrocyte phenotype.

Since the cell cycle regulators, *p21, p57*, and *Ccnd1* have been implicated as important regulators of chondrocyte proliferation and hypertrophic differentiation, we further measured their gene expression in the presence and absence of Notch activation. Interestingly, while no significant variation was observed in the amounts of *p21* after over-expression of NICD1, expression of *p57* was significantly induced by NICD1. In contrast, *Ccnd1* expression was significantly decreased in NICD transfected cells (Fig. 1A). These results suggest *p57* maybe a potential target of Notch signaling, which could play an important role during Notch induced chondrocyte differentiation. To further determine how cell cycle progression is affected by Notch activation, cell viability (7-AAD) and proliferation (BrdU) were analyzed by flow cytometry (Fig. 1B,C). Although no significant change was observed in G2/M cell populations, our data indeed showed a large increase of G0/G1 cell populations in NICD transfected cells when compared to control cells. Furthermore, a significant decrease of the S phase cell population was observed in NICD transfected cells indicating that Notch activation induces cell cycle exit from S-phase during chondrocyte differentiation.

### BMP-2 induced chondrocyte hypertrophy is inhibited by Notch signaling inactivation

**Figure 2:**
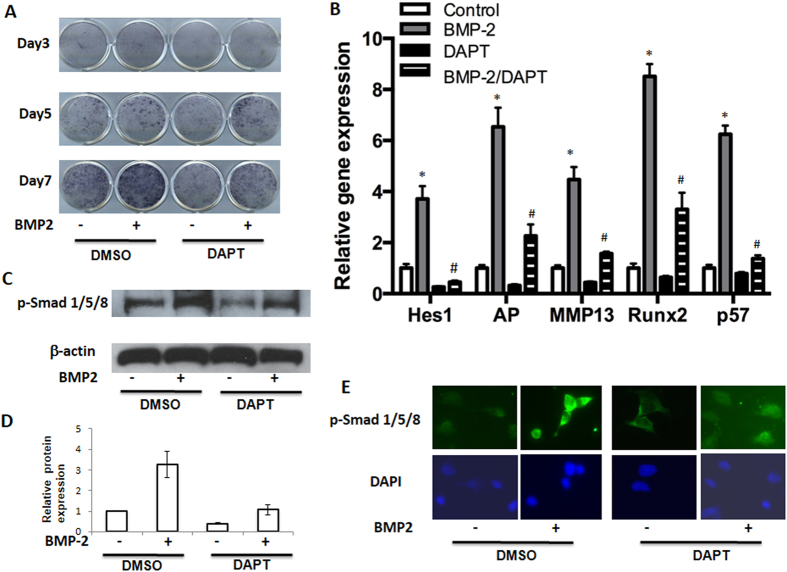
Notch inhibition ablates BMP-induced chondrocytes maturation. Primary mouse sternal chondrocytes were cultured in regular media and treated with DAPT (5 uM) and/or BMP2 (100 ng/ml) before being harvested for AP staining, total RNA and protein isolation at the indicated time points. (**A**) BMP treatment resulted in significant increases in AP staining at days 3, 5, and 7, compared to vehicle-treated controls. (**B**) Real time PCR showed a markedly decreased expression of *Hes1, AP, MMP13, Runx2, p57* in chondrocyte after treatment with DAPT for 7 days. (**C**) Western blot analysis of p-SMAD1/5/8 expression in chondrocytes after 6 hours of DAPT and/or BMP treatment. β-actin was used as a loading control. (**D**) Quantization of band density of western blot results showed the folds over control. (**E**) Immunofluorescence data showed an increased expression of p-SMAD1/5/8 in nuclear area in BMP2 treated cells and a decreased labeling in both peri-nuclear and nuclear areas in DAPT treated cells. PCR data are means ± SD of three independent experiments performed in duplicate and the control gene expression level was set at 1. (*p < 0.05 compared with control at same time point; ^#^p < 0.05 compared with DAPT alone cells).

Previously we demonstrated that a small molecule inhibitor of the gamma-secretase complex (DAPT) that blocks all Notch signaling resulted in delayed chondrocyte hypertrophy^8^. Here we analyzed the effects of DAPT on BMP-induced chondrocyte differentiation. In this experiment, cultures of primary mouse sternal chondrocytes were treated with BMP2 and/or DAPT for 7 days. Consistent with previous findings, control cultures showed a normal progression in AP staining (a surrogate for chondrocyte hypertrophy) from days 3 to 7, while BMP2 treatment significantly enhanced AP staining in day 5 and 7 treated cultures. Interestingly, DAPT treated cells showed decreased AP staining in both control cultures and BMP2 treated cultures at most time-points (Fig. 2A).

We further investigated the effects of DAPT on the expression of regulators and markers of chondrocyte hypertrophy. In day 7 cultures, *Runx2, Mmp13 and Alp* gene expression was significantly enhanced by BMP treatment, whereas DAPT treatment mildly reduced their expression in the absence of BMP2 and abolished their activation in the presence of BMP2 (Fig. 2B). These findings indicate that activation of Notch signaling is essential for BMP-induced chondrocyte hypertrophy in primary chondrocytes.

To determine whether cell cycle regulators were also involved in this process, we measured gene expression of *p57* in the presence of BMP activation and/or Notch inhibition. Real-time RT-PCR results showed a significant increase of *p57* gene expressions by the addition of BMP2. DAPT not only inhibited endogenous *p57* expression, but also abrogated BMP2 induced gene expression (Fig. 2B). Finally, expression of Notch target gene *Hes1* was also measured to confirm that Notch signaling was inhibited in these cells by DAPT treatments (Fig. 2B).

Since phosphorylated SMAD 1/5/8 (p-SMAD 1/5/8) is a key signaling event following BMP receptor activation, we next investigated the effect of DAPT on the phosphorylation of SMAD 1/5/8 by Western blot. DAPT-treated cells initially showed a decrease in p-SMAD 1/5/8 relative to controls, and BMP-induced p-SMAD 1/5/8 was inhibited by DAPT (Fig. 2C,D). These data suggest that the down-regulation of BMP signaling via Notch inhibition occurs in part through the regulation of BMP receptor signaling and SMAD 1/5/8 phosphorylation. To determine if this inhibition is directly regulated by DAPT, we measured p-SMAD 1/5/8 protein levels using immunofluorescence at 2 hours post-treatment of BMP2 and DAPT (Fig. 2E). In control cells, most of the endogenous p-SMAD 1/5/8 localized to the cytoplasm and nuclear area, while a rapid increase of nuclear p-SMAD 1/5/8 was observed as early as 2 hours after BMP treatment. In contrast, a quick decrease of p-SMAD 1/5/8 in the nucleus was observed in DAPT treated cells, and BMP-induced up-regulation of p-SMAD 1/5/8 was also largely inhibited by treatment of DAPT. These results suggest there is a possible direct regulation between the Notch and BMP signaling pathways.

### Down-regulation of SMAD 1/5/8 impairs Notch-induced chondrocyte hypertrophy

**Figure 3:**
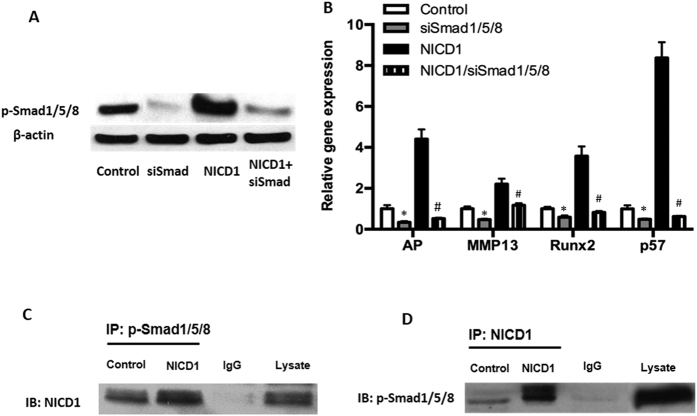
Knockdown of SMAD1/5/8 impairs Notch-induced chondrocytes differentiation. ATDC5 cells were transfected with SMAD1/5/8 siRNA oligos or NICD1 plasmids for 48 hours before being harvested for western blot and RT-PCR analysis. (**A**) A drastic reduction in SMAD1/5/8 protein expression was seen in cells that had SMAD1/5/8 knocked down. (**B**) SMAD1/5/8 knocking down significantly inhibited *AP, Runx2, MMP13, p57* gene expression and resulted in the reduction of NICD1-induced gene expression. (**C**) Immunoprecipitation (IP) by p-SMAD1/5/8 antibody showed an interaction between p-SMAD1/5/8 and NICD1. (**D**) Immunoprecipitation by anti-NICD1 antibody showed a strong interaction between SMAD1/5/8 and NICD1. Normal lgG serving as negative control. Data B are means ± SD of three independent experiments performed in duplicate and the control gene expression level was set at 1. (*p < 0.05 compared with control groups; ^#^p < 0.05 compared with NICD1 alone cells).

Since BMP signaling has been reported to induce *p57* in osteoblastic cells through activation of p-SMAD 1/5/8^17^, we investigated whether p-SMAD 1/5/8 signaling mediates the Notch induction of *p57* expression in chondrocytes. In this experiment, we used siRNA to knock down *Smad 1/5/8* gene expression in NICD plasmid transfected cells. Western blot analysis confirmed an efficient down-regulation of p-SMAD 1/5/8 protein levels in *siSMAD 1/5/8* transfected cells. Interestingly, NICD1-induced p-SMAD 1/5/8 expression was also dramatically reduced (Fig. 3A). Real-time RT-PCR data further showed that transfecting chondrocytes with the *SMAD 1/5/8* siRNA resulted in a significant inhibition of NICD-induced expression of *Alp, Runx2 and Mmp13* (Fig. 3B). More importantly, knocking down SMAD 1/5/8 dramatically reduced *p57* expression in NICD transfected cells (Fig. 3B) providing evidence that sustained Notch signaling induces chondrocyte cell cycle arrest through *p57* induction, which is primarily dependent on the levels of p-SMAD 1/5/8.

Although cross-talk between the BMP and Notch signaling pathways is known to exist in different cellular contexts^18^, it remains unclear whether the physical interaction of NICD and SMAD 1/5/8 proteins exist in maturing chondrocytes. To explore the possible interaction between NICD and p-SMAD 1/5/8, co-immunoprecipitation was performed using both NICD and p-SMAD 1/5/8 antibodies in cells transfected with or without NICD1 plasmids. In control cells, a weak interaction between NICD1 and SMAD 1/5/8 was detected. In contrast, NICD1 over expression resulted in a strong association between NICD1 and p-SMAD 1/5/8 proteins in chondrocytes (Fig. 3C,D) confirming the direct interaction and regulation between the BMP and Notch signaling pathways during chondrocyte differentiation.

### PPM1A is involved in Notch-induced SMAD 1/5/8 phosphorylation within chondrocytes

**Figure 4:**
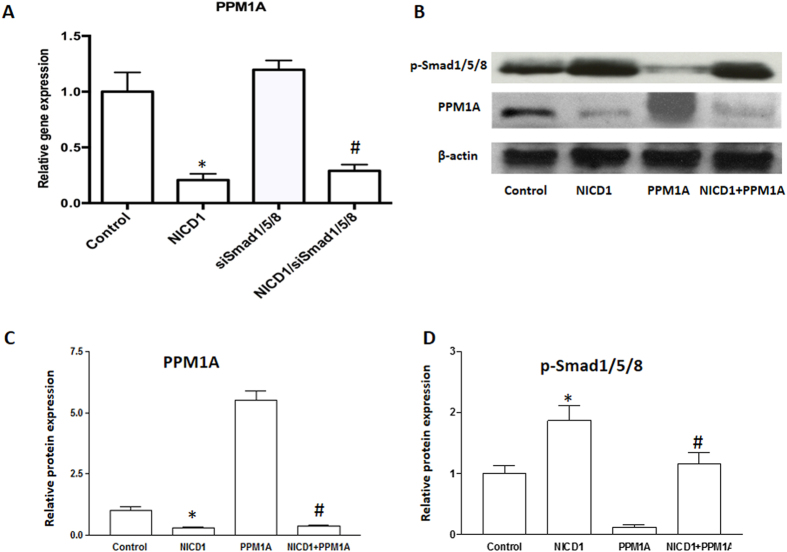
Notch activation decreased the expression of PPM1A. ATDC5 cells were transfected with siRNA oligos or plasmids for 48 hours before being harvested for western blot and PCR analysis. (**A**) PPM1A gene expression was measured by Real time RT-PCR in NICD1 expressing and/or SMAD1/5/8 knocking down ATDC5 cells after culture for 48 hours. (**B**) p-SMAD1/5/8 and PPM1A protein levels were measured by Western blot analysis in ATDC5 cells transfected with NICD1 and/or PPM1A plasmids. (**C**) Quantization of band density of PPM1A in western blot results. (**D**) Quantization of band density of p-SMAD1/5/8 in western blot results. PCR data are means ± SD of three independent experiments performed in duplicate and the control expression level was set at 1. (*p < 0.01 compared with vehicle control; ^#^p < 0.05 compared with siSMAD1/5/8 or PPM1A alone cells).

Multiple phosphatases have been proposed to catalyze the removal of phosphates from the SXS motif of BMP-SMADs, including pyruvate dehydrogenase phosphatase (PDP), small C-terminal domain phosphatases (SCPs) and Protein Phosphatase 1A (PPM1A). Among these, PPM1A was reported as a key SMAD phosphatase to terminate phospho-SMAD 1/5/8 activity by increasing dephoshorylation or proteasomal degradation^19^,^20^. We next asked whether PPM1A is also involved in Notch-induced SMAD 1/5/8 phosphorylation. To answer this question, we first analyzed *PPM1A* expression in both control and NICD1 transfected cells with and without *SMAD 1/5/8* siRNA. We demonstrate that *PPM1A* was significantly inhibited by NICD1 in ATDC5 cells, and its expression was not affected by down-regulating SMAD 1/5/8 (Fig. 4A). We further transfected ATDC5 cells with NICD1 and/or PPM1A plasmids. After 48 hours of culture, total protein was harvested for Western blot analysis (Fig. 4B). Reduced protein levels of p-SMAD 1/5/8 was observed in cells with overexpression of PPM1A, indicating significant SMAD 1/5/8 de-phosphorylation and inhibition of BMP signaling in these cells. Consistent with the data shown in Fig. 3A, NICD significantly increased p-SMAD 1/5/8 protein expression. Interestingly, PPM1A expression in PPM1A transfected cells was also inhibited by co-transfection of NICD1, and down-regulation of p-SMAD 1/5/8 by PPM1A was reversed by overexpression of NICD1 (Fig. 4C,D) suggesting Notch signaling is a stronger inhibitor of PPM1A expression in chondrocytes. Overall, these results indicate that activation of Notch signaling enhances chondrocyte maturation and hypertrophy by increasing or prolonging cellular p-SMAD 1/5/8 levels via inhibition of PPM1A expression and activity.

## Discussion

Although both BMP and Notch signaling play similar roles in chondrocyte hypertrophy and maturation, it remains unclear how they interact to control this process at the molecular and cellular levels. Since BMP signaling regulation of chondrocyte differentiation largely depends on SMAD 1/5/8 activity^21^ and BMP signaling is known to stimulate the expression of cyclin-dependent kinase inhibitors via the SMAD pathway during osteoblast differentiation^17^, it is plausible that Notch signaling may also regulate chondrocyte proliferation and differentiation in a similar manner. In this study, we show that over-expression of NICD1 in a chondrogenic cell line significantly induced expression of *Alp, Runx2, and Mmp13*. At the same time, the cyclin-dependent kinase inhibitor, *p57*, was also significantly increased suggesting that cell cycle arrest is involved in Notch signaling induced chondrocyte hypertrophic differentiation. AP staining further showed inhibition of Notch signaling by DAPT was sufficient to ablate BMP2-induced chondrocyte hypertrophy. RT-PCR analysis further confirmed these results by showing reduced expression of *Runx2* and *Mmp13* in DAPT treated cells. More importantly, BMP2 induced p-SMAD 1/5/8 levels were significantly reduced by DAPT treatments. These results argue strongly that Notch activation is partially required for maintaining active BMP signaling during chondrocyte maturation and hypertrophy. In addition, when SMAD 1/5/8 levels were directly reduced in chondrocytes, NICD induced expression of chondrocyte maturation markers were significantly reduced further demonstrating that Notch signaling controls chondrocyte differentiation at least in part by regulating BMP signaling.

Cell cycle regulation is important for chondrocyte hypertrophic differentiation, in which a withdrawal from cell proliferation allows pre-hypertrophic chondrocytes to properly differentiate into hypertrophic chondrocytes. Two CDKI gene families have been identified as key regulators for cell cycle progression. The first is the INK4 gene family that encodes p16, p15, p18, and p19, all of which bind to CDK4 and CDK6 and inhibit their kinase activities by interfering with their association with D-type cyclins^22^. The second is Cip/Kip family, including p21^23^,^24^,^25^, p27^26^,^27^ and p57^28^,^29^, which bind to both CYCLIN and CDK subunits and can modulate the activities of CYCLIN D-, E-, A-, and B-CDK complexes^22^. In this study, we found that Notch signaling not only induced cell-cycle inhibitor *p57* expression during chondrocyte differentiation, but also induced cell cycle transition from S phase to G0/G1 phase. These results suggest that Notch-induced chondrocyte maturation could be initiated through inhibition of cell cycle progression. Based on these results, we hypothesize that with the activation of Notch signaling, chondrocytes are no longer maintained in a proliferative state, choosing instead to exit the cell cycle and proceed through differentiation that may be further regulated by other signaling cues.

Chondrocyte transition from proliferation to differentiation is also regulated by several paracrine factors, among which BMP acts as both a positive regulator of proliferation and promotes the initiation of chondrocyte hypertrophic differentiation^30^. Previous studies demonstrate that both p21 and p57 are involved in BMP signaling-induced chondrogenic differentiation, in which expression of p21 was induced by up-regulation of BMP2 in skeletal progenitor cell micromass cultures^31^, and although p57 alone was not sufficient for driving chondrocyte hypertrophy, but was capable of augmenting BMP2 induced maturation^32^. Despite these findings, how BMP-2 acts with other signaling factors to regulate cell cycle arrest and chondrocyte hypertrophy is still unknown. Since Notch signaling is mostly active in prehypertrophic and hypertrophic chondrocytes^33^ and induces *p57* expression, here we speculate that Notch may act synergistically with BMP signals to induce enough p57 expression to shift chondrocytes out of the proliferative phase and into hypertrophy. Indeed, we observed a significant increase of *p57* expression during BMP2 induced chondrocyte maturation and hypertrophy, and this increase was further abrogated by inhibition of Notch signaling supporting the possible synergistic effects for both Notch and BMP signaling in chondrocyte maturation.

To gain additional insights into the cellular interactions of Notch and BMP signaling in the transition of mitotic chondrocytes to a mature post mitotic fate, we further assayed the effects of the BMP signaling key effector SMAD 1/5/8 on chondrocyte hypertrophy following Notch activation. Our data show that although Notch signaling induced *p57* expression, this induction was significantly decreased upon knocking down SMAD 1/5/8, indicating that Notch induction of *p57* is largely dependent on SMAD 1/5/8 expression and activity. Consistent with this view, co-immunoprecipitation shows a direct interaction between NICD and SMAD1/5/8, confirming cross-talk of the BMP and Notch signaling pathways during chondrocyte differentiation.

**Figure 5:**
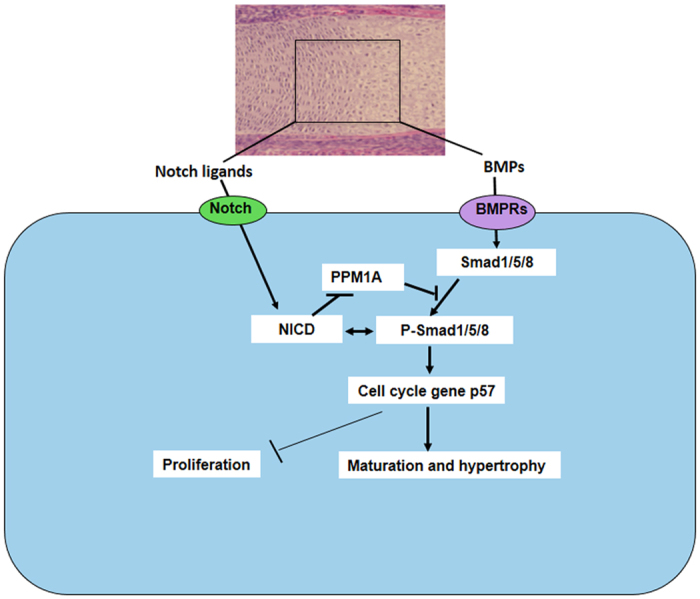
Models of potential Notch/BMP signaling crosstalk during chondrocyte maturation. During growth plate development, Notch and BMP signaling begins with the binding of ligands (Jagged1, BMP2 *et al.*) and receptors, the Notch intracellular domain NICD stabilizes BMP signaling key factor, p-SMAD1/5/8 by inhibition of PPM1A activity. Either NICD or p-SMAD1/5/8 induces cell cycle exit by up-regulation of p57 leading to decreased proliferation and increased differentiation in mature chondrocytes. Arrows indicate induction and perpendicular lines indicate suppression.

Additionally, our data showed a significant decrease of PPM1A expression in NICD overexpressing cells, and this decrease was not affected by knocking down SMAD 1/5/8, suggesting that Notch reinforces BMP/SMAD 1/5/8 signaling via the inhibition of PPM1A and thereby prolongs and strengthens SMAD transcriptional outputs. Co-transfection experiments further confirmed this regulation by showing a reversal of SMAD 1/5/8 expression by NICD1 in PPM1A expressing cells. Since the possible contribution of other phosphatases to the regulation of SMAD 1/5/8 expression was not investigated in this study; we cannot eliminate the possibility that Notch signaling induced p-SMAD1/5/8 up-regulation via alternative phosphatases. Based on these findings, we have demonstrated that Notch signaling controls chondrocyte maturation at least in part via the regulation of both BMP signaling and cell cycle transition (Fig. 5).

Taken together, the present study identified at least one key and understudied mechanism by which Notch signaling regulates chondrocyte proliferation and differentiation. Activation of Notch signaling induces p-SMAD1/5/8 via the repression of PPM1A, increases cell cycle inhibitor p57 gene expression, and promotes chondrocyte cell cycle arrest (Fig. 5).

## Materials and Methods

### Cell culture

Chondrogenic ATDC5 cells were obtained from the Riken Cell Bank (Tsukuba, Japan). ATDC5 cells were cultured in medium consisting of a 1:1 mixture of Dulbecco’s modified Eagle’s medium (DMEM) and nutrient mixture F-12 (DMEM/F-12; Invitrogen, Carlsbad, CA, USA) containing 50μg/ml ascorbic acid (Roche Molecular Biochemicals, Mannheim, Germany) and 10% (v/v) fetal bovine serum, at 37 °C in a humidified atmosphere of 5% CO_2_, as described previously^34^. Animal studies were performed in accordance with appropriate guidelines. All experimental protocol were approved by the Louisiana State University Committee on Animal Resources (Protocol # P-15-005). Primary chondrocytes were isolated from the ribcages of 3-day-old mouse pups (C56BL/6J) and initially cultured as previously described^35^. Cells were treated one day after plating with either 5 μM DAPT (*N*-[*N*-(3, 5-difuorophenacetyl)-L-alanyl]-*S*-phenylglycine *t*-butyl ester), and/or BMP2 (100 ng/ml; Peprotech, Inc., Rockyhill, NJ) in complete media and allowed to differentiate and mature for up to 7 days. Alkaline phosphatase (AP) staining and real-time RT-PCR was performed as previously described^36^. Primer sequences for *AP, Runx2, Mmp13, Sox9, p21, p57, NICD1, Hes1* and *β-actin* are listed in Table 1.

### Flow Cytometry analysis

Cell cycle analysis was carried out using BD Pharmingen FITC BrdU Flow Kit. Briefly, 70–80% confluence ATDC5 chondrocytes in 6-well plates were first transfected with NICD1 plasmid. After 48 hours of culture, BrdU was added to all wells with final concentration of 10 μM of BrdU in media for an additional 6-hour incubation. Cells were harvested and fixed with BD Biosciences Cytofix/Cytoperm buffer, then treated with DNase for 1 h at 37 °C. Cells were stained with FITC-conjugated anti-BrdU Antibody for 20 min at room temperature, followed by 7-AAD for 10 min. Flow cytometry was performed in 8-color BD FACS Canto-II, and cell cycle was analyzed using FlowJo software.

### Immunofluorescence analysis

For immunofluorescence, ATDC5 chondrocytes were plated at 1,000 cells/cm^2^ on coverslips and grown for 24 hours in normal medium. At 60% confluence, cells were serum deprived for 6 hours and treated with DAPT and/or BMP for 2 hours. Cells were then fixed in 4% paraformaldehyde in PBS for 20 minutes at room temperature and permeabilized with 0.3% Triton X-100 in PBS for 10 minutes. Cells were washed in PBS and incubated with 0.5% BSA dissolved in PBS at room temperature for 20 minutes. Cultures were then incubated for 2 hours at room temperature with rabbit anti-SMAD1/5/8 (Santa Cruz Biotechnology) diluted 1:50 in PBS. After washing with PBS, cells were incubated with FITC conjugated anti-rabbit IgG for 1 hour at room temperature. Reaction controls were performed using a non-immune rabbit immunoglobulin IgG, or by omitting the primary antibody. Cover slips were mounted on slides with PBS/glycerol (1:1), and slides were imaged by fluorescent microscopy using a Zeiss Axioplan microscope.

### Cell Transfection assay

The construct encoding the mouse Notch NICD1 gene and 3XFLAG tag, cloned into the mammalian expression vector CMV-7, was a gift from Dr. Kopan (Washington University School of Medicine, St. Louis, Missouri, USA.). *PPM1A* gene, cloned into the mammalian expression vector pcDNA3.1, was a gift from Dr. Chen (Rush University Medical Center, Chicago, USA.). ATDC5 chondrocytes were plated at a density of 5.0 × 10^5^ cells/well in 6-well plates 1 day before transfection at 70–80% confluence. Transfections were performed using Lipofectamine^TM^ 2000 (Invitrogen, Gaithersburg, MD) according to the manufacturer’s recommendations. Forty-eight hours post-transfection, the cells were harvested for protein and RNA isolation. For siRNA transfection, ATDC5 cells at 90% confluence were transfected with 25nM *SMAD 1/5/8* small interfering RNA (siRNA), or control non-targeting siRNA (Applied Biosystems/Ambion, Denmark) with or without NICD plasmids using Lipofectamine^TM^ RNAiMax. In all experiments, control vectors were used to keep the total amount of transfected DNA identical. After 48 hours of transfection, cells were harvested for real-time RT-PCR and western blot as described^18^.

### Western blot and immunoprecipitation analysis

40μg of total protein was used for Western blot analysis. The samples were separated using 10–15% SDS–PAGE. These proteins were then transferred to polyvinylidene difluoride (PVDF, Millipore) membranes. After blocking with 5% non-fat milk, the PVDF membranes were incubated with primary antibodies in blocking buffer overnight at 4 °C. On the following day, PVDF membranes were incubated with the appropriate secondary antibodies for 2 hours at room temperature. After the membranes had been soaked in an enhanced chemiluminescence reagent (Thermo Scientific) for 5 minutes, the blots were visualized using X-ray film. Primary antibodies for PPM1A, anti-NICD1 and p-SMAD 1/5/8 (Cell Signaling Technology) were used in this analyses and β-ACTIN (sigma) antibody was used as a loading control. For the immunoprecipitation assay, 5 μg of either anti-NICD1 or Phospho-SMAD1/5/8 antibody was added to 500μg proteins extracted from control ATDC5 cells or NICD1 plasmid transfected cells in RIPA buffer (25 mM Tris-HCl, pH7.6, 150 mM NaCl, 1% Nonidet P-40, 1% sodium deoxycholate). Overnight incubation at 4 °C allowed complexes to form, after which 20 μl of 50% slurry protein A/G-agarose beads (Santa Cruz Biotechnology) was added and incubated at 4 °C for 3 hours. Immunoprecipitates were washed four times in RIPA buffer and analyzed by Western blots.

### Real-time PCR analysis

Total RNA was isolated from cell culture using RNeasy Mini Kit from Qiagen Inc. One microgram of RNA was subjected to reverse transcription using the iScript cDNA synthesis Kit (Bio-Rad). The obtained cDNA was then amplified via real-time RT-PCR using an ABI 7500 Real-time PCR System (Applied Biosystems) and SYBR^®^ Green Real time PCR Supermix (Bio-Rad). The primers used for real-time RT-PCR are listed in Table 1, and β*-actin* was used as the housekeeping gene. Quantification of the relative expression levels of these target genes was achieved by normalizing to β-actin using the ΔΔCt method.

### Statistical analysis

The above experiments were repeated at least three times independently. All the data were presented as mean ± SD. Statistical significance among the groups was assessed using one-way ANOVA. The level of significance was P < 0.05.

## Additional Information

**How to cite this article**: Shang, X. *et al.* Notch signaling indirectly promotes chondrocyte hypertrophy via regulation of BMP signaling and cell cycle arrest. *Sci. Rep.* **6**, 25594; doi: 10.1038/srep25594 (2016).

## Figures and Tables

**Table 1:** Mouse Primers Used for Real-Time RT-PCR Experiments.

	Forward primer	Reverse primer	Accession number
Runx2	GCATGGTGGAGGTACTAGCTG	GCCGTCCACTGTGATTTTG	NM009821
AP	ACTGGTAAGTGGGGCAAGAC	CCACACCAAATTCCTGTTCA	BC052326
MMP13	AGGAAGCTGGCAGACCAGTA	CGTTCTTCACCGACT TCCTC	AF421878
CcnD1	TGGAGAAGGTACTTACGGTGTGG	TGGGCACTCCTTCTTCCTCGCT	NM007659
P27	CAAGATCGCATCTCCCGGC	CTGACAAGCCACGCAGTAGA	NM006396
Sox9	AGGAAGCTGGCAGACCAGTA	CGTTCTTCACCGACTTCCTC	AF421878
P57	CCAGCGATACCTTCCCAGTG	CCTCGAAGAGGCACATCCTG	NM1161624
NICD1	GGAGGCATCCTACCCTTTTC	TGTGTTGCTGGAGCATCTTC	NM010928
Hes1	TCAACACGACACCGGACAAA	CCTTCGCCTCTTCTCCATGAT	NM008235
β-actin	AGATGTGGATCAGCAAGCAG	GCGCAAGTTAGGTTTTGTCA	NM007393
